# KMnCuTe_2_: a layered antiferromagnetic semiconductor with long metal–metal distance†

**DOI:** 10.1039/d2ra04789f

**Published:** 2022-10-11

**Authors:** Fan Sun, Zhao Liu, Jiawei Lin, Jun Deng, Zhongnan Guo, Wenxia Yuan

**Affiliations:** Department of Chemistry, School of Chemistry and Biological Engineering, University of Science and Technology Beijing Beijing 100083 China guozhongn@ustb.edu.cn wxyuanwz@163.com; Research & Development Centre for Functional Crystals, Beijing National Laboratory for Condensed Matter Physics, Institute of Physics, Chinese Academy of Sciences Beijing 100190 China

## Abstract

The magnetic semiconductor in a two-dimensional system is a major subject for both theoretical and experimental investigations. Here we report the synthesis of a new quaternary manganese chalcogenide KMnCuTe_2_, which shows layered structure and antiferromagnetic (AFM) semiconducting features. Single crystals of KMnCuTe_2_ were obtained using a self-flux method and based on single-crystal X-ray diffraction, KMnCuTe_2_ adopts the ThCr_2_Si_2_-type structure composed of edge-sharing tetrahedral layers separated by K^+^ cations. The Mn and Cu atoms randomly distribute in the centre of tetrahedral units. Attributed to the large radius of Te, KMnCuTe_2_ has large lattice parameters (*a* = 4.3115(3) Å and *c* = 14.9360(20) Å), leading to a long metal–metal distance (3.049 Å) in the tetrahedral layers. Based on the experiments and theoretical calculations, KMnCuTe_2_ exhibits a G-type AFM interaction with the transition temperature at around 225 K and an indirect semiconducting nature with the band gap of 0.95 eV. The magnetic semiconducting property of KMnCuTe_2_ is unique in AMnMCh_2_ systems (A = Li, Na, K, M = Cu, Ag and Ch = S, Se, Te), which could be associated with the large metal–metal distance. Our work not only highlights the role of metal–metal interactions on regulating the properties of ThCr_2_Si_2_-type compounds, but also provides a feasible strategy to obtain the layered magnetic semiconductor.

## Abstract 2

We synthesized a new quaternary telluride KMnCuTe_2_ with long metal–metal distance in tetragonal layers, which shows an indirect band gap of 0.95 eV and long-range antiferromagnetic ordering.

## Graphical abstract

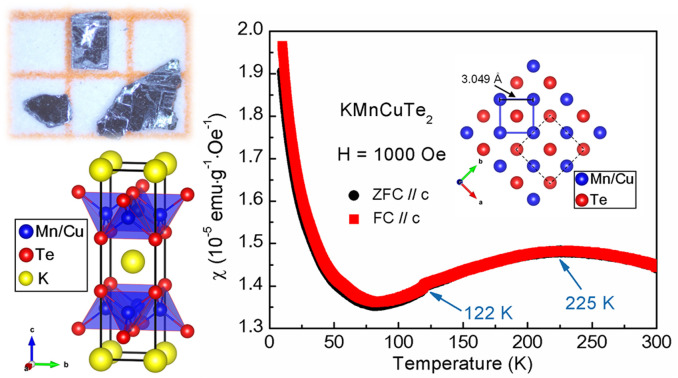

## Introduction

The ThCr_2_Si_2_-type structure is a very common one for ternary inorganic compounds. In the last decade, the transition metal chalcogenides with ThCr_2_Si_2_-type structure have attracted intense attention attributed to the discovery of Fe-based high-temperature superconductors.^1–3^ From the crystal structure point of view, the ThCr_2_Si_2_-type iron chalcogenides are composed of edge-sharing FeCh_4_ tetrahedral layers (Ch = S, Se, Te) with the alkali metal intercalated in between the layers.^4^ Stimulated by the fascinating Fe-based ThCr_2_Si_2_-type compounds, the Ni- and Co-based analogues have also been fully investigated, and exhibit heavy-fermion behavior and long-range magnetic order, respectively.^5–9^ Interestingly, the ThCr_2_Si_2_-type structure can also be formed in transition metal chalcogenides by mixing the metal site in tetrahedral layers, largely enriching the compositional diversity of this material family. In these “metal-mixed” phases, the transition metal ions are partially substituted by monovalent metal ions M^+^, forming a series of quaternary metal chalcogenides including AFeMCh_2_, ACoMCh_2_, AMnMCh_2_, ACrMCh_2_ and AZnMCh_2_ (A = K, Rb, Cs, M = Cu, Ag, Li and Ch = S, Se, Te).^10–24^ Recently, the alkaline-earth metal chalcogenides KMgCuSe_2_ and KMgCuTe_2_ with ThCr_2_Si_2_-type structure were also synthesized by our group.^25^ Given the similar chemical composition with the Fe-based high-temperature superconductors, the KFeAgTe_2_ was described in more details focusing on the interactions between its crystal structure, magnetic order and electronic structure.^12,13,26^ Very recently, the intertwined magnetic order and nematic orders, which were considered as the feature of Fe-based high-temperature superconductors, were observed in KFeAgTe_2_.^27^ A exotic spin-nematic state induced by a small strain was also observed in this layered iron telluride.^28^

The layered manganese chalcogenide AMnMCh_2_ (A = K, Rb, Cs, M = Cu, Ag and Ch = S, Se, Te) are also intriguing materials, not only as a similar specimen to Fe-based superconductors in electronic structure but also as a potential two-dimensional (2D) magnetic system. It was reported that some members in AMnMCh_2_ family show the spin-glass state while the others are paramagnets.^16–18^ However, the long-range magnetic order such as antiferromagnetic (AFM) interaction has not been reported yet in this Mn-based quaternary family. It has been revealed that the lattice parameters, especially the metal–metal distances in *ab* plane, play a key role on determining the magnetism and transport properties for ThCr_2_Si_2_-type materials.^18,22^ Synthesizing the new member in AMnMCh_2_ family with large metal–metal distance could be expected to realize the unique physical property.

In this work, we report the single-crystal growth of a new member in AMnMCh_2_ family, KMnCuTe_2_. It is indicated that the title compound adopts the ThCr_2_Si_2_-type structure that is comprised of edge-sharing [Mn(Cu)Te_4_] tetrahedral layers with intercalated K^+^ cations. The Mn and Cu atoms randomly distribute in the centre of tetrahedron with the ratio of Mn : Cu close to 1 : 1 due to the similar radii of Mn and Cu. Due to the large radius of Te, KMnCuTe_2_ has much larger lattice parameters (*a* = 4.3115(3) Å and *c* = 14.9360(20) Å) compared to the sulfides and selenides in AMnMCh_2_, leading to a long metal–metal distance in tetrahedral layers (3.049 Å). Based on the experiments and theoretical calculations, we demonstrate that KMnCuTe_2_ exhibits unique magnetic semiconducting behavior with an indirect band gap (0.95 eV) and G-type AFM interaction.

## Experimental section

### Synthesis

Single-crystals of KMnCuTe_2_ were grown by self-flux method. Powders of Mn (99.99%, Aladdin), Cu (99.9%, Aladdin) and Te (99.9%, Aladdin) were mixed with 1 : 1 : 2 molar ratio and ground in an agate mortar. The raw materials were cold-pressed into disks with 10 mm diameter under 200 kg cm^−2^ uniaxial stress and put into the alumina crucibles together with K ingot (97%, SINOPHARM) as predetermined compositions. The total mass of the samples was around 2 g. The alumina crucibles were sealed into the quartz tubes under vacuum and then firstly heated to 473 K and held for 12 hours for the pre-reaction of potassium and Te. After pre-reaction, the samples were re-ground and cold-pressed again. All the manipulations were carried out inside an argon-filled glove box (O_2_ < 1 ppm) in order to prevent the sample oxidation. The disks were heated to 1273 K in 12 hours, held for 48 hours, and then cooled down to 874 K with the cooling rate of 4 K per hour before cooling to room temperature. It should be mentioned that the crucibles was shook for several times during the heating process of synthesis to obtain the homogeneous phase.

### Characterization

Powder X-ray diffraction (PXRD) were collected on a PANalytical diffractometer (X'Pert PRO MRD) equipped with Cu Kα radiation (*λ* = 1.5406 Å) operation at 40 kV and 40 mA. Single crystal X-ray diffraction (SCXRD) was performed on a Bruker D8 Venture diffractometer at 50 kV and 1.4 mA with Mo Kα radiation (*λ* = 0.71073 Å). All data were collected at room temperature under nitrogen flow. The crystal structure of KMnCuTe_2_ was solved (using direct method) and refined using Jana 2006 package.^29^ Morphology of the samples was investigated *via* scanning electron microscopy (SEM, Hitachi S-4800), and the component analysis was made by energy dispersive X-ray spectroscopy (EDS). The result for each sample was obtained based on the average of 10 sets of data. The magnetization susceptibility was measured using a vibrating sample magnetometer (VSM, Quantum Design) from 300 to 10 K. The electrical resistivity of single crystal samples was measured *via* four-probe method using a physical property measurement system (PPMS, Quantum Design). The specific heat capacity was also measured using PPMS. The optical diffuse reflectance measurements were performed at room temperature using a Shimadzu UV-3600 UV-vis-NIR spectrometer and BaSO_4_ was used as the reference of 100% reflectance. The absorption data were calculated based on the obtained reflectance spectrum according to the Kubelka–Munk equation, *α*/*S* = (1 − *R*)^2^/(2*R*), where *R* is the reflectance and *α* and *S* are the absorption and scattering coefficients, respectively.^30^

### Calculation

All calculations were performed in the density functional theory (DFT) using Vienna *ab into* simulation package (VASP), and the projector-augmented wave (PAW) scheme and Perdew–Burke–Ernzerhof generalized gradient approximation (PBE-GGA) were used for the exchange correlation functional.^31–33^ The wave functions were expanded into plane-wave up to a cutoff energy of 450 eV.^34^ We built a 2 × 2 × 1 supercell to simulate the different magnetic structures, and a Monkhorst–Pack *k*-mesh of 4× 4 × 2 was used for sampling the first Brillouin zone in the self-consistent calculation.^35^ The structures were relaxed with an energy convergence criterion of 10^−6^ eV, ensuring that the maximum force on an atom was <0.03 eV Å^−1^. Considering the strong correlations of 3d electrons of Cu and Mn, on-site Hubbard U for 3d orbitals with *U* = 5 eV was adopted.^36^

## Results and discussion

**Fig. 1:**
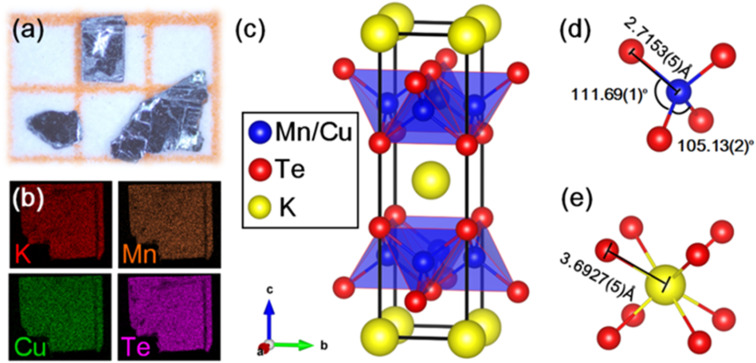
(a) Optical photograph of the single-crystal sample of KMnCuTe_2_. (b) Elemental mapping of the sample. (c) Crystal structure of KMnCuTe_2_ with tetragonal *I*4/*mmm* space group. The detailed bond lengths and angles in (d) [Mn(Cu)Te_4_] tetrahedron and (e) [KTe_8_] cube.

We successfully synthesized a new quaternary manganese chalcogenide KMnCuTe_2_, which is a new member in AMnMCh_2_ (A = K, Rb, Cs, M = Cu, Ag and Ch = S, Se, Te) family.^16–18^ Using self-flux method, single crystal sheets were obtained by cleaving the crystal grains as shown in Fig. 1a, and the products show black color and are about 1 mm in size. Elemental mapping in Fig. 1b confirms the homogeneous distribution of K, Mn, Cu and Te in sample. Chemical analysis by EDS shows that the atomic ratio of K : Mn : Cu : Te is 0.97 : 0.96 : 0.98 : 2, which is close to the stoichiometry.

The single-crystal of KMnCuTe_2_ was picked directly from the product for SCXRD measurement. The obtained crystallographic information and structural parameters are listed in Tables S1–S3.† It can be seen that the final refinement gives very low weight agreement factors *R*_int_, *R* and *R*_w_, suggesting that our structure solution is satisfactory. KMnCuTe_2_ crystallizes in the ThCr_2_Si_2_-type structure, which adopts a body-centered tetragonal lattice with the space group of *I*4/*mmm* (No. 139). The lattice parameters are refined to be *a* = 4.3115(3) Å and *c* = 14.9360(20) Å, comparable to the analogous tellurides including KFeCuTe_2_, KFe_0.85_Ag_1.15_Te_2_, CsMn_1.18_Ag_0.64_Te_2_ and CsFe_0.72_Ag_1.28_Te_2_.^12,20,23,24^ As shown in Fig. 1c, the structure shows very typical layered nature, which is composed of the edge-sharing [Mn(Cu)Te_4_] tetrahedral layer extending in the *ab* plane and the potassium ions are located in the [KTe_8_] cubic sites between the layers. The Mn and Cu atoms are randomly distributed in the centre of tetrahedral units (shown in Fig. 1c) and this mixed occupation is mainly due to the similar radii of Mn and Cu. The [Mn(Cu)Te_4_] tetrahedron is compressed along the *ab* plane with the four Te–Mn(Cu)–Te angles across the *ab* plane being 111.69(1)° and the other two Te–Mn(Cu)–Te angles on the same side being 105.13(2)°, as shown in Fig. 1d. The Mn(Cu)–Te bond length is refined as 2.7153(5) Å, which is larger than Fe(Cu)–Te bond length (2.6344(8) Å) in the analogue, KFeCuTe_2_.^20^ The distance between K^+^ and the neighboring Te^2−^ is 3.6927(5) Å (Fig. 1e), implying the negligible interaction between the alkali metals and the transition telluride layers. The refined composition from SCXRD is KMn_0.96_Cu_1.04_Te_2_, which is in good agreement with the EDS result. The PXRD pattern of the powder sample from grinding the crystals matches well with the simulated one from SCXRD, as shown in Fig. S1.† We found that KMnCuTe_2_ is chemically stable in ambient condition, with the PXRD pattern basically unchanged after exposure in air for 7 days (Fig. S1†).

The crystal structure of KMnCuTe_2_ is also related to that of the BaM_*x*_Te_2_ system (M = Cu and Ag) reported by Jana *et al.*^37^ Both KMnCuTe_2_ and BaM_*x*_Te_2_ are composed of the edge-sharing tetrahedral layers, in which the Mn(Cu)–Te bond length is slightly smaller than the Cu–Te bonds in BaCu_0.43_Te_2_ (2.72–2.74 Å).^37^ Different from the alternating stacking of K^+^ and [Mn(Cu)Te_4_] layers in KMnCuTe_2_, the [Cu_*x*_Te_4_] tetrahedral layers are separated by both Ba^2+^ cations and Te square planes in BaCu_*x*_Te_2_. A modulated structure was also observed in BaM_*x*_Te_2_ due to the distorted Te square nets, but no modulated structure has been found in AMnMCh_2_ system yet.

**Fig. 2:**
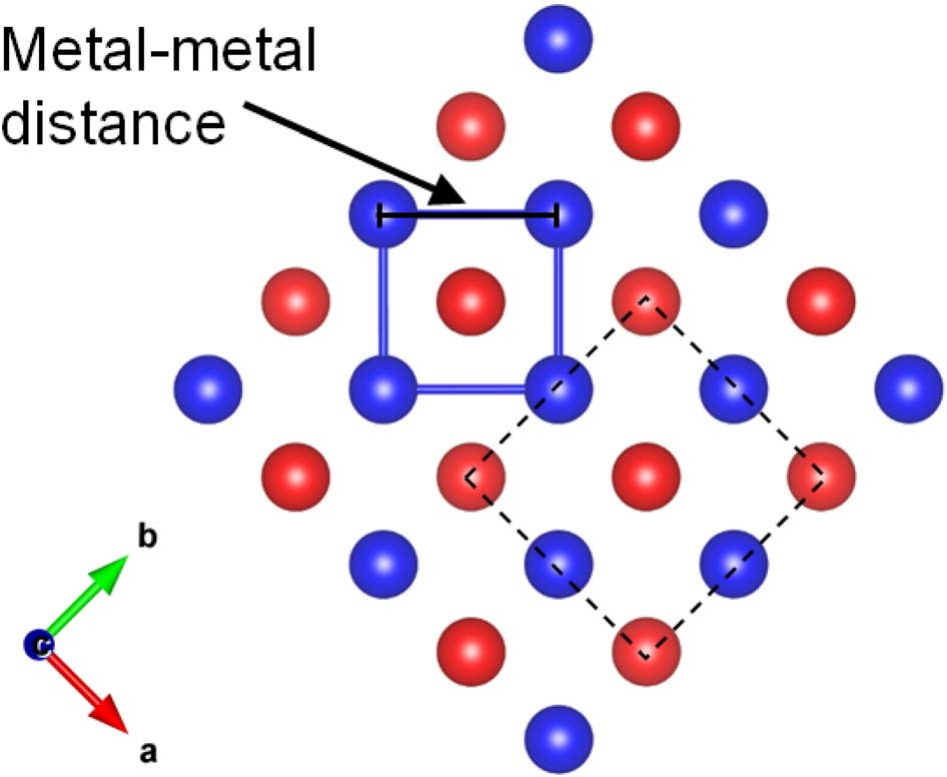
The metal–metal distance along the layer direction in ThCr_2_Si_2_-type compounds. The dash square is the unit cell of the structure.

As we mentioned, KMnCuTe_2_ is a new member in AMnMCh_2_ family, and ascribing to the large radius of Te, both *a* and *c* lattice constants of KMnCuTe_2_ are much larger than those of the sulfides and selenides in AMnMCh_2_, as shown in Table 1. Only the reported CsMn_1.18_Ag_0.64_Te_2_ shows larger lattice parameters than KMnCuTe_2_, but the related physical property was not described.^23^ The metal–metal distance in *ab* plane, which equals to 
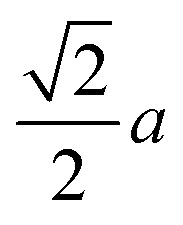
, is also listed in Table 1. The Mn(Cu)–Mn(Cu) distance is 3.049 Å in KMnCuTe_2_ (Fig. 2), which is much longer that those in KMnCuSe_2_ (2.893 Å) and KMnCuS_2_ (2.800 Å),^16,17^ and is also longer than the Mn(Ag)–Mn(Ag) bond in KMnAgSe_2_ (3.004 Å).^18^ This demonstrates a very weak interaction between the metal atoms in tetrahedral layers in KMnCuTe_2_. It has been widely believed that the structure parameter, especially the metal–metal distance, plays an important role on the magnetic and transport properties of AMnMCh_2_ compounds. With the relatively large metallic distance in layer, KMnCuTe_2_ provides an important platform to understand the structure–property relationship of AMnMCh_2_ materials, which will be discussed in details below.

**Table 1:** Structure parameters and magnetic behavior of KMnCuTe_2_ compared to the other AMnMCh_2_ in previous works (SG = spin-glass state, PM = paramagnetism and AFM = antiferromagnetism)

Composition	*a* (Å)	*c* (Å)	Metal–metal distances (Å)	MT	Ref.
KMnCuS_2_	3.9596(6)	13.297(3)	2.800	SG	16
RbMnCuS_2_	4.0001(3)	13.624(2)	2.828	SG	16
CsMnCuS_2_	4.0514(3)	14.147(2)	2.865	SG	16
KMnCuSe_2_	4.0912(8)	13.893(4)	2.893	SG	17
RbMnCuSe_2_	4.1283(6)	14.169(3)	2.919	PM	17
CsMnCuSe_2_	4.1902(5)	14.686(3)	2.963	PM	17
KMnAgSe_2_	4.2486(3)	13.943(2)	3.004	SG	18
**KMnCuTe_2_**	**4.3115(3)**	**14.9360(20)**	**3.049**	**AFM**	**This work**
CsMn_1.18_Ag_0.64_Te_2_	4.520(2)	15.484(8)	3.196	Not mentioned	23

**Fig. 3:**
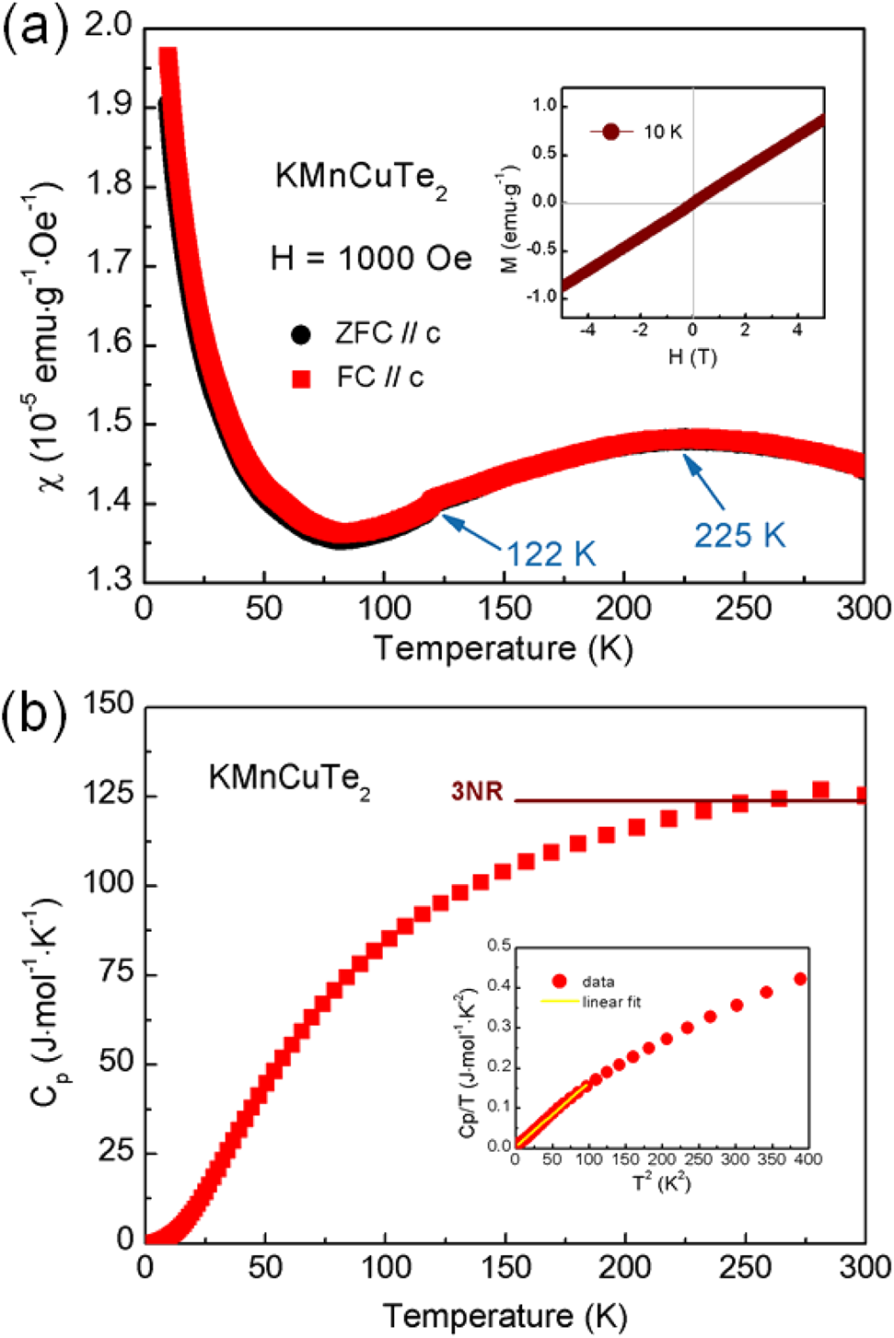
(a) Temperature dependence of magnetic susceptibility for KMnCuTe_2_ single crystals from 300 to 10 K with the applied field (*H* = 1000 Oe) parallel to the *c* axis. Inset shows field dependence of magnetic susceptibility at 10 K. (b) Temperature dependence of specific heat capacity *C*_p_ of KMnCuTe_2_ from 300 to 2 K. The inset shows the relation between *C*_p_/*T* and *T*^2^ in low temperature range (<20 K) with the linear fitting plotted as yellow line.

To investigate the influence of the large lattice parameters on magnetic properties, we performed the measurements on temperature dependent magnetic susceptibility (*M*–*T* curve) of KMnCuTe_2_. As shown in Fig. 3, the magnetic susceptibility shows a broad transition at around 225 K, suggesting a low-dimensional AFM interaction. Meanwhile, a kink was also observed at about 122 K in the *M*–*T* curve. In high temperature range, the magnetic susceptibility deviates from the Curie–Weiss law, implying the complex magnetic interactions between Mn^2+^ ions at high temperatures. The absence of the bifurcate between the zero-field cooling (ZFC) and field cooling (FC) curves in low temperature range rules out the spin-glass behavior, different from magnetic states of KMnCuSe_2_ and KMnCuS_2_. It is worth noting that although this low-dimensional AFM ordering is unique in AMnMCh_2_ family, the similar magnetic behavior was observed in some layered transition metal oxychalcogenides such as Ba_2_CoO_2_Ag_2_Se_2_.^38^ The Curie–Weiss-like upturn in low temperature range is presumably due to the paramagnetic impurities on the surface of KMnCuTe_2_ crystal. The magnetization loop at 10 K is also shown in inset of Fig. 3a, which exhibit linear dependence on the field (*H*) with basically no hysteresis, further confirming the AFM ordering in this compound.

In our previous work, it was observed that the magnetism in Fe-based AFeMCh_2_ family is mainly determined by the *a* lattice constant, where the long-range AFM order can only be maintained with the *a* axis larger than 4.19 Å while the spin-glass behavior turns to appear with the *a* axis smaller than this critical value.^22^ Interestingly, the AFM interaction observed in KMnCuTe_2_ demonstrates the similar regulation rule of the *a* lattice parameter over the magnetic behavior of AMnMCh_2_ compounds with the critical value as ∼4.3 Å (Table 1), implying the uniform mechanism of the structural modulation on magnetic coupling in ThCr_2_Si_2_-type material family with different transition metals.

The heat capacity *versus T* (*C*_p_–*T* curve) was plotted in Fig. 3b. However, there is no clear anomaly observed in *C*_p_–*T* curve at the AFM transition temperature (225 K). This phenomenon could be corresponding to the broad transition in *M*–*T* curve and suggests that the magnetic entropies above transition temperature decreases gradually during the cooling process. As shown in the inset of Fig. 3b, the *C*_p_(*T*)/*T versus T*^2^ shows a non-linear characteristic at low temperature (below 20 K). By fitting the data below 10 K with the expression: *C*_p_/*T* = *γ* + *βT*^2^, where *γ* is the Sommerfeld coefficient, the fitted *γ* and *β* were obtained as 3.35 mJ mol^−1^ K^−2^ and 1.60 mJ mol^−1^ K^−4^, respectively. According to the formula *θ*_D_ = (12π^4^NR/*β*)^1/3^, the Debye temperature *θ*_D_ is estimated to be about 182 K.

**Fig. 4:**
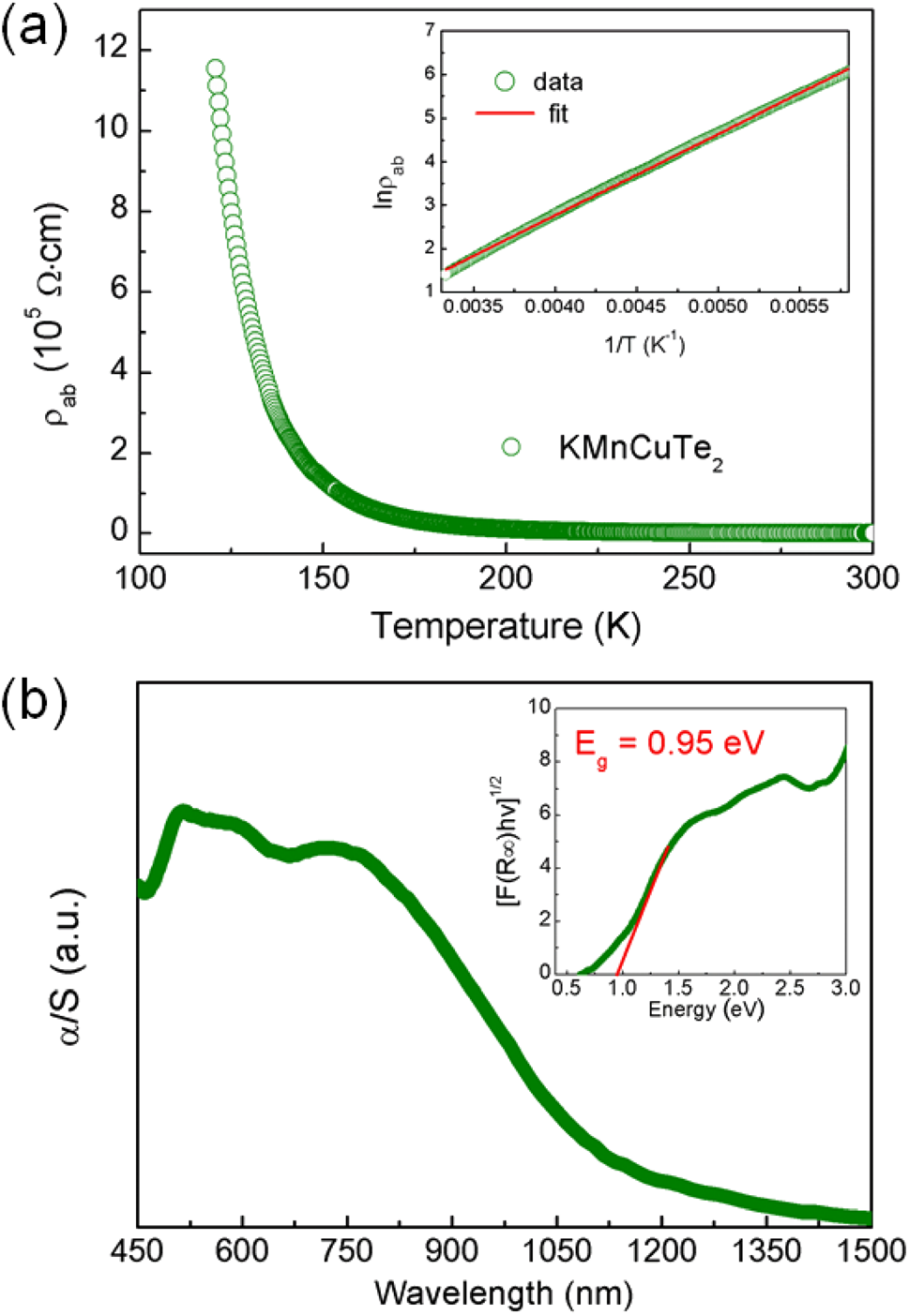
(a) Temperature dependence of the in-plane resistivity for KMnCuTe_2_ single crystal from 300 to 120 K. Inset shows the fitted result for the data in high temperature range from 300 to 150 K using the thermal activation model. (b) UV-vis diffuse reflectance spectrum of KMnCuTe_2_. Inset shows the extracted indirect band gap according to Tauc plot.

The variation of in-plane resistivity with temperature for KMnCuTe_2_ crystal is shown in Fig. 4a. Clearly it exhibits a semiconducting behavior from 120 K to 300 K. The resistivity data obey the thermally activated behavior *ρ* = *ρ*_0_ exp(*E*_a_/*k*_B_*T*) as shown in inset, and the activation energy *E*_a_ is calculated as 0.16 eV. The electrical resistivity at room temperature is around 408 Ω cm, which is much larger than those of KMnCuSe_2_ and KMnCuS_2_. It is generally believed that the telluride shows smaller resistivity than its analogous selenide and sulfide due to the smaller electronegativity of Te. We indicate that the abnormal rules in KMnCuTe_2_ compared to KMnCuSe_2_ and KMnCuS_2_ could be resulted from the long metal–metal distance, which weakens the metallic orbital overlap between Mn(Cu) atoms and significantly increases the in-plane resistivity. It is noticed that in-plane resistivity of KMnCuTe_2_ is also larger than those of KFeCuTe_2_ and KFeAgTe_2_, suggesting the significant effect of transition metal species on electrical properties of ThCr_2_Si_2_-type compounds.^12,13,20^

The UV-vis diffuse reflectance spectrum was collected to show the optical property of KMnCuTe_2_ crystals. To extract the band gap, the reflectance data were transformed to pseudo-absorption data using Kubelka–Munk equation as described in the experimental section (Fig. 4b). The optical band gap was extracted using the indirect model as suggested by the band structure calculation described below, and the obtained band gap (*E*_g_) is 0.95 eV, which is consistent with the black color of the crystal. Hence it can be concluded that KMnCuTe_2_ is an antiferromagnetic semiconductor with the narrow band gap.

**Fig. 5:**
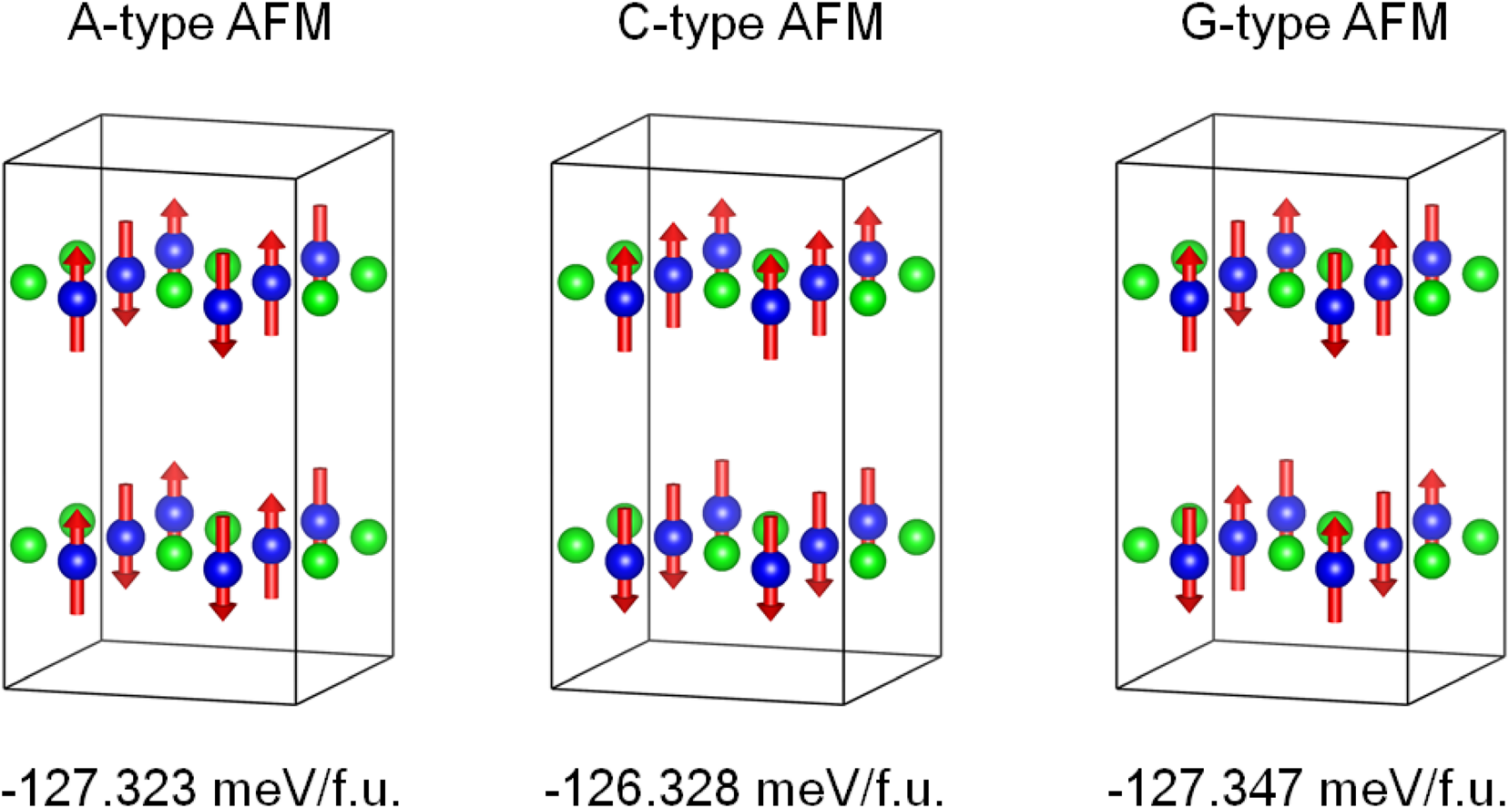
The calculated energies of different AFM structures of KMnCuTe_2_ in a 2 × 2 × 1 supercell, showing that the G-type AFM is the energy favorable ground state of KMnCuTe_2_.

**Fig. 6:**
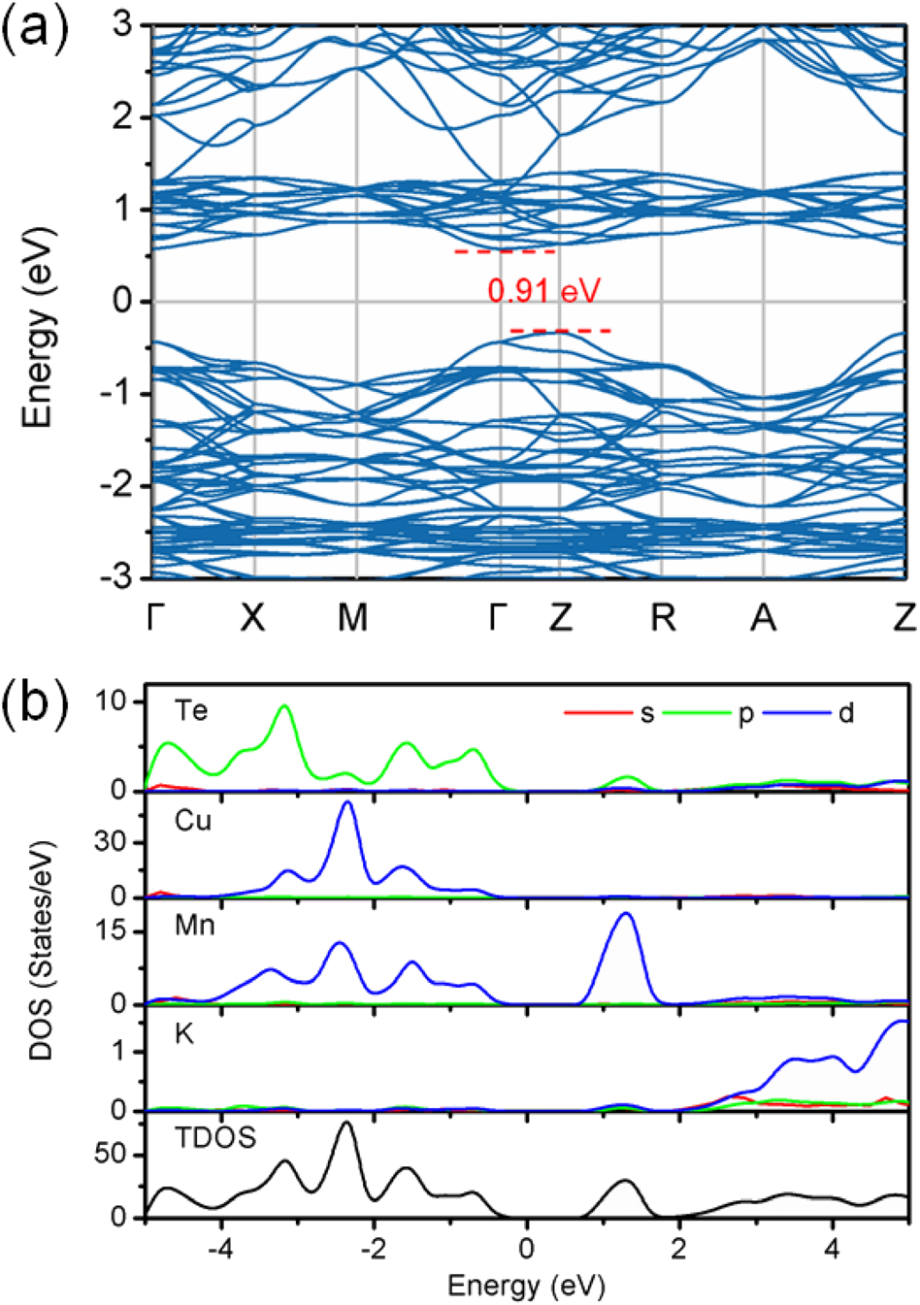
(a) The calculated electronic band structure and (b) density of states for KMnCuTe_2_.

The density functional theory calculations were also carried out to better understand the nature of magnetism and electronic structure of KMnCuTe_2_. Three AFM structures, including A-type, C-type and G-type were built in a 2 × 2 × 1 supercell as shown in Fig. 5, and the calculation suggested the G-type AFM as the energy favorable ground state of KMnCuTe_2_. It should be mentioned that the energy difference to the A-type AFM state is only 0.024 meV per f.u., suggesting that the A-type AFM can also be the possible ground state of KMnCuTe_2_. With the G-type AFM structure, the electronic band structure and density of states of KMnCuTe_2_ were calculated. As shown in Fig. 6a, the valence band maximum (VBM) is located at *Z* point while the conduction band minimum (CBM) is located at *Γ*, indicating that KMnCuTe_2_ is an indirect semiconductor. The calculated band gap is 0.91 eV, in consistent with the experimental result (0.95 eV). Based on the partial electronic density of states (DOS) shown in Fig. 6b, the valence band of KMnCuTe_2_ near the Fermi level (*E*_F_) is contributed by the orbitals of Mn, Cu and Te, while the conduction band near the band edge is mainly composed of the 3d orbitals of Mn.

## Conclusion

Here we report a new quaternary chalcogenide KMnCuTe_2_ which exhibits layered structure and antiferromagnetic semiconducting behavior. As a new member in AMnMCh_2_ (A = K, Rb, Cs, M = Cu, Ag and Ch = S, Se, Te) material family, KMnCuTe_2_ adopts the ThCr_2_Si_2_-type structure with mixed Mn and Cu occupation. The refined lattice parameters (*a* = 4.3115(3) Å and *c* = 14.9360(20) Å) and resulted metal–metal distance (3.049 Å) are larger than the sulfides and selenides in AMnMCh_2_. KMnCuTe_2_ shows a long-range G-type antiferromagnetic interaction with the transition temperature at around 225 K, very unique in the AMnMCh_2_ system. The resistivity and diffuse reflectance spectra indicate that KMnCuTe_2_ is an indirect semiconductor with the optical band gap as 0.95 eV. These results shed light on the relationship between crystal structure and physical properties of ThCr_2_Si_2_-type materials.

## Conflicts of interest

The authors declare no competing financial interest.

## Supplementary Material

RA-012-D2RA04789F-s001

RA-012-D2RA04789F-s002
