# Supplementary material for: KMnCuTe2: a layered antiferromagnetic semiconductor with long metal–metal distance
Source: RSC Adv. 2022 Oct 11;12(45):29003–9. doi: 10.1039/d2ra04789f (PMC9552607; doi:10.1039/d2ra04789f)
Supplement: RA-012-D2RA04789F-s001 [file RA-012-D2RA04789F-s001.pdf]

Electronic Supplementary Material (ESI) for RSC Advances.
This journal is © The Royal Society of Chemistry 2022

Supplementary Information

$KMnCuTe_2$: a layered antiferromagnetic semiconductor with long metal-metal distance

Fan Sun,[a] Zhao Liu,[a] Jiawei Lin,[a] Jun Deng,[b] Zhongnan Guo,[a*] and Wenxia Yuan [a**]

[a] *Department of Chemistry, School of Chemistry and Biological Engineering, University of Science and Technology Beijing, Beijing 100083, China.*

E-mail :guozhongn@ustb.edu.cn wxyuanwz@163.com;

Fax:+86-010-62333033; Tel:+86-010-62332221

[b] *Research & Development Center for Functional Crystals, Beijing National Laboratory for Condensed Matter Physics, Institute of Physics, Chinese Academy of Sciences, Beijing 100190, China.*

Table S1. Crystal data and structure refinement for $KMnCuTe_2$ at 293 K.

Empirical formula	$KMn_{1.04}Cu_{0.96}Te_2$
Formula weight	412.52
Temperature	293(2) K
Wavelength	0.71073 Å
Crystal system	tetragonal
Space group	*I*4/*mmm*
Unit cell dimensions	a = 4.3115(3) Å<br>c = 14.9360(20) Å
Volume	277.64(10) $Å^3$
Z	2
Density (calculated)	4.935 g/cm^3
Absorption coefficient	16.932 mm^{-1}
F(000)	354
Crystal size	0.4 × 0.3 × 0.05 mm^3
θ range for data collection	2.73 to 27.95°
Index ranges	$-5 \leqslant h \leqslant 5$, $-5 \leqslant k \leqslant 5$, $-19 \leqslant l \leqslant 19$
Reflections collected	1097
Independent reflections	130 [R_{int} = 0.0389]
Completeness to θ = 25.242°	99%
Refinement method	Full-matrix least-squares on F^2
Data / restraints / parameters	130 / 0 / 9
Goodness-of-fit	1.57
Final R indices [I > 2σ(I)]	R_{obs} = 0.0202, wR_{obs} = 0.0477
R indices [all data]	R_{all} = 0.0206, wR_{all} = 0.0478
Largest diff. peak and hole	1.15 and -0.46 $e \cdot Å^{-3}$

$R = \Sigma||F_o|-|F_c|| / \Sigma|F_o|$, $wR = \{\Sigma[w(|F_o|^2 - |F_c|^2)^2] / \Sigma[w(|F_o|^4)]\}^{1/2}$ and $w=1/[\sigma^2(Fo^2)+(0.0317P)^2]$ where $P=(Fo^2+2Fc^2)/3$

Table S2. Atomic coordinates ($\times 10^4$) and equivalent isotropic displacement parameters ($Å^2 \times 10^3$) for $KMnCuTe_2$ at 293 K with estimated standard deviations in parentheses.

Label	x	y	z	Occupancy	U_{eq}^*
K(1)	0	0	0	1	33(1)
Te(1)	0	0	3605(1)	1	19(1)
Mn(1)	0	5000	2500	0.52(3)	25(1)
Cu(1)	0	5000	2500	0.48(3)	25(1)

$^*U_{eq}$ is defined as one third of the trace of the orthogonalized U_{ij} tensor.

Table S3. Anisotropic displacement parameters ($Å^2 \times 10^3$) for $KMnCuTe_2$ at 293 K with estimated standard deviations in parentheses.

Label	U_{11}	U_{22}	U_{33}	U_{12}	U_{13}	U_{23}
K(1)	30(2)	30(2)	38(2)	0	0	0
Te(1)	19(1)	19(1)	20(1)	0	0	0
Mn(1)	23(1)	23(1)	30(1)	0	0	0
Cu(1)	23(1)	23(1)	30(1)	0	0	0

The anisotropic displacement factor exponent takes the form: $-2\pi^2[h^2a^{*2}U_{11} + ... + 2hka^*b^*U_{12}]$.

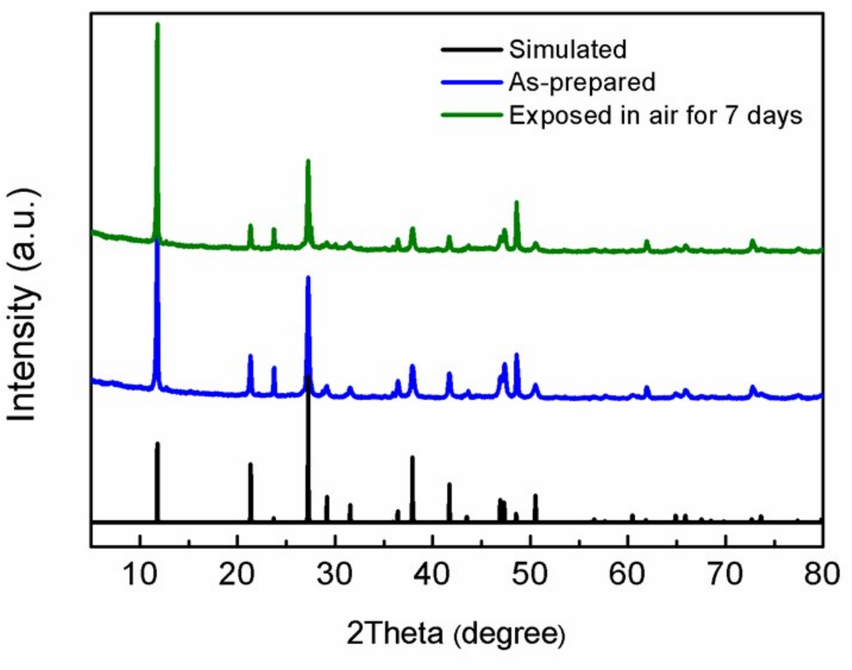

Fig. S1 PXRD patterns of the $KMnCuTe_2$ powder sample compared with the simulated one from SCXRD.
